# Complete mitochondrial genome and phylogenetic analysis of the sea anemone *Heteractis doreensis* (Quoy & Gaimard, 1833)

**DOI:** 10.1080/23802359.2026.2627019

**Published:** 2026-02-09

**Authors:** Yanling Liao, Yirong Ye, Wenping Wang, Jinxing Fu, Xueying Zhang, Bingmiao Gao, Tianle Tang

**Affiliations:** Engineering Research Center of Tropical Medicine Innovation and Transformation of Ministry of Education, Hainan Provincial Key Laboratory for Research and Development of Tropical Herbs, College of Pharmacy, Hainan Medical University, Haikou, China

**Keywords:** Sea anemone, *Heteractis doreensis*, mitochondrial genome, phylogenetic

## Abstract

The first complete mitochondrial genome of *Heteractis doreensis* (Quoy & Gaimard, 1833) was sequenced to clarify its mitogenomic features and phylogenetic position. The circular genome is 20,720 bp long with 60.34% A+T content, comprising 13 protein-coding genes, two ribosomal RNA genes, two transfer RNA genes, and one putative open reading frame. Maximum-likelihood phylogenetic analysis of 19 Hexacorallia species revealed that *H. doreensis* clusters with *Stichodactyla haddoni* and *Heteractis crispa* in a strongly supported clade (bootstrap = 96%) within Stichodactylidae. These findings provide valuable molecular insights into the evolution and phylogeography of *H. doreensis*, supporting future research on its conservation and breeding.

## 1. Introduction

**Figure 1.**
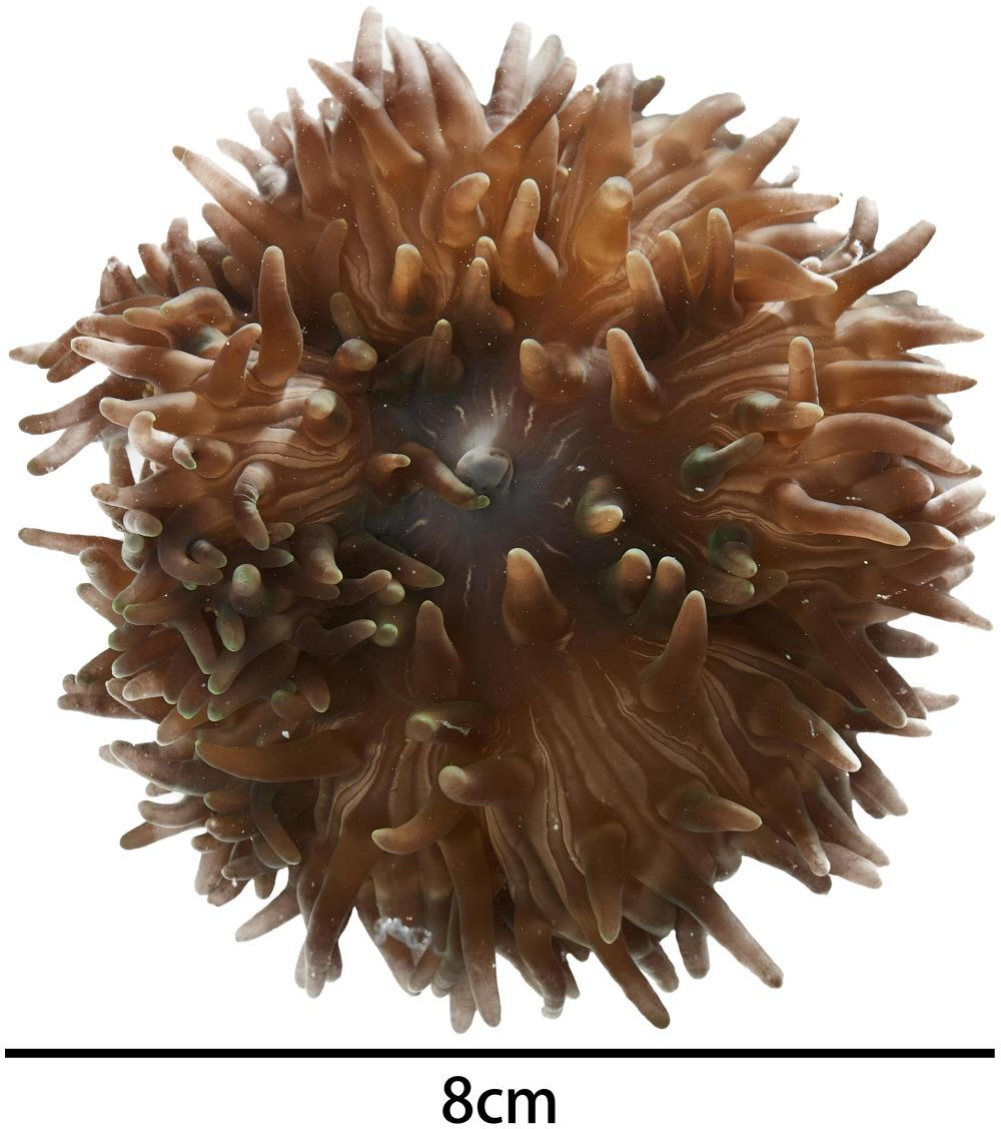
Reference image of *H. doreensis* taken by Bingmiao Gao.

*Heteractis doreensis* (Figure 1) is a sea anemone belonging to the order Actiniaria, suborder Enthemonae, family Stichodactylidae, and genus *Heteractis* (Daly et al. 2016). Sea anemones are widely distributed from tropical to deep-sea environments, including depths exceeding 10,000 m, and produce a diverse array of peptide and protein toxins critical for prey capture, defense, and interspecific competition (Madio et al. 2017, 2018; Menezes and Thakur 2022). These bioactive compounds have emerging applications in psychopharmacology and medical drug development (O’Hara et al. 2021). High-throughput omics approaches have identified numerous sea anemone peptide neurotoxins (Kim et al. 2017). The mitochondrial genome is characterized by maternal inheritance, compact size, and relatively rapid evolution. Its replication is regulated by nuclear-encoded factors. Since mitochondrial DNA is abundant and amenable to PCR-based amplification, complete mitochondrial genomes are widely used in molecular systematics. Despite their ecological and pharmacological significance, genomic resources for *H. doreensis* are limited. Therefore, sequencing and comparative analysis of the mitochondrial genome of *H. doreensis* offers an opportunity to clarify the species’ evolutionary characteristics and phylogenetic position. Research on Actiniaria phylogeny has addressed taxonomy, nomenclature, identification, and biodiversity issues (Sinniger et al. 2007; Oliveira et al. 2012; Stampar et al. 2022). With advances in sequencing technologies, whole mitochondrial genomes have become an increasingly powerful tool for resolving phylogenetic relationships within Cnidaria (Kayal et al. 2015; Novosolov et al. 2022; González Muñoz et al. 2023; Li et al. 2023). This study determined the complete mitochondrial genome of *H. doreensis* and resolved its phylogenetic placement. These data enhance our understanding of the genetic diversity and evolutionary relationships within Stichodactylidae and provide a molecular reference for taxonomic resolution, comparative genomics, and conservation planning.

## 2. Materials and methods

*H. doreensis* specimens were collected from the Xisha Islands, South China Sea (16.34° N, 111.49° E) on 17 May 2021. A specimen was deposited at the Key Laboratory of Tropical Translational Medicine, Ministry of Education, Hainan Medical University (Bingmiao Gao, gaobingmiao@hainmc.edu.cn), Haikou, Hainan, China under voucher number CHMU0267. Species identity was determined based on morphological characteristics, including size, coloration, and basal column morphology, and was confirmed by BLAST analysis through comparison of the sequencing results with reference sequences in the NCBI database.

The complete mitochondrial genome was extracted using the Column mtDNAout kit (Tianda, Beijing, China). The sample concentration and purity were assessed using a NanoDrop™ Spectrophotometer (Thermo Fisher Scientific, Waltham, MA) and Qubit^®^ 2.0 Fluorometer (Life Technologies, Waltham, MA), respectively. Qualified DNA samples were then subjected to Illumina standard library preparation (San Diego, CA), and a paired-end sequencing library with an insert size of approximately 350 bp was constructed. Following library preparation, quality control was performed using quantitative PCR and an Agilent 2100 Bioanalyzer (Agilent Technologies, Santa Clara, CA). Finally, DNA libraries that met the quality criteria were sequenced on an Illumina NovaSeq 6000 high-throughput platform (Illumina, San Diego, CA) using a PE150 sequencing strategy. Evaluation of the sequencing data quality and coverage revealed an average depth of 58.74× across the mitochondrial genome (Supplementary Figure 1).

The obtained clean reads were assembled *de novo* using SPAdes (version 3.5.0) (Bankevich et al. 2012). Genome annotation was performed using MITOS (Bernt et al. 2013) and open reading frame Finder (Cheng et al. 2013), while transfer RNA (tRNA) genes were specifically annotated using ARWEN (Laslett and Canbäck 2008) and tRNAscan-SE (Lowe and Chan 2016). Phylogenetic analyses were performed based on the complete mitochondrial genome of *H. doreensis* and 18 species across the subclass Hexacorallia (Beagley et al. 1998; Foox et al. 2016; Zhang and Zhu 2017; Chi et al. 2018; Surm et al. 2019; Fu et al. 2021; Johansen et al. 2021), including Actiniidae, Halcampoididae, Haloclavidae, Nyantheae, Phymanthidae, Sagartiidae, Stichodactylidae, and Zoantharia. Evolutionary analyses were conducted in MEGA11 (Tamura and Nei 1993), and the evolutionary history was inferred using the maximum-likelihood (ML) method with the Tamura–Nei substitution model (Tamura et al. 2021). Initial heuristic trees were generated automatically by applying the Neighbor-Joining and BioNJ algorithms to a pairwise distance matrix estimated under the Tamura–Nei model, followed by the selection of a topology with the highest log-likelihood value. Branch support was evaluated using 1000 bootstrap replicates (Barroso et al. 2025).

## 3. Results

**Figure 2.**
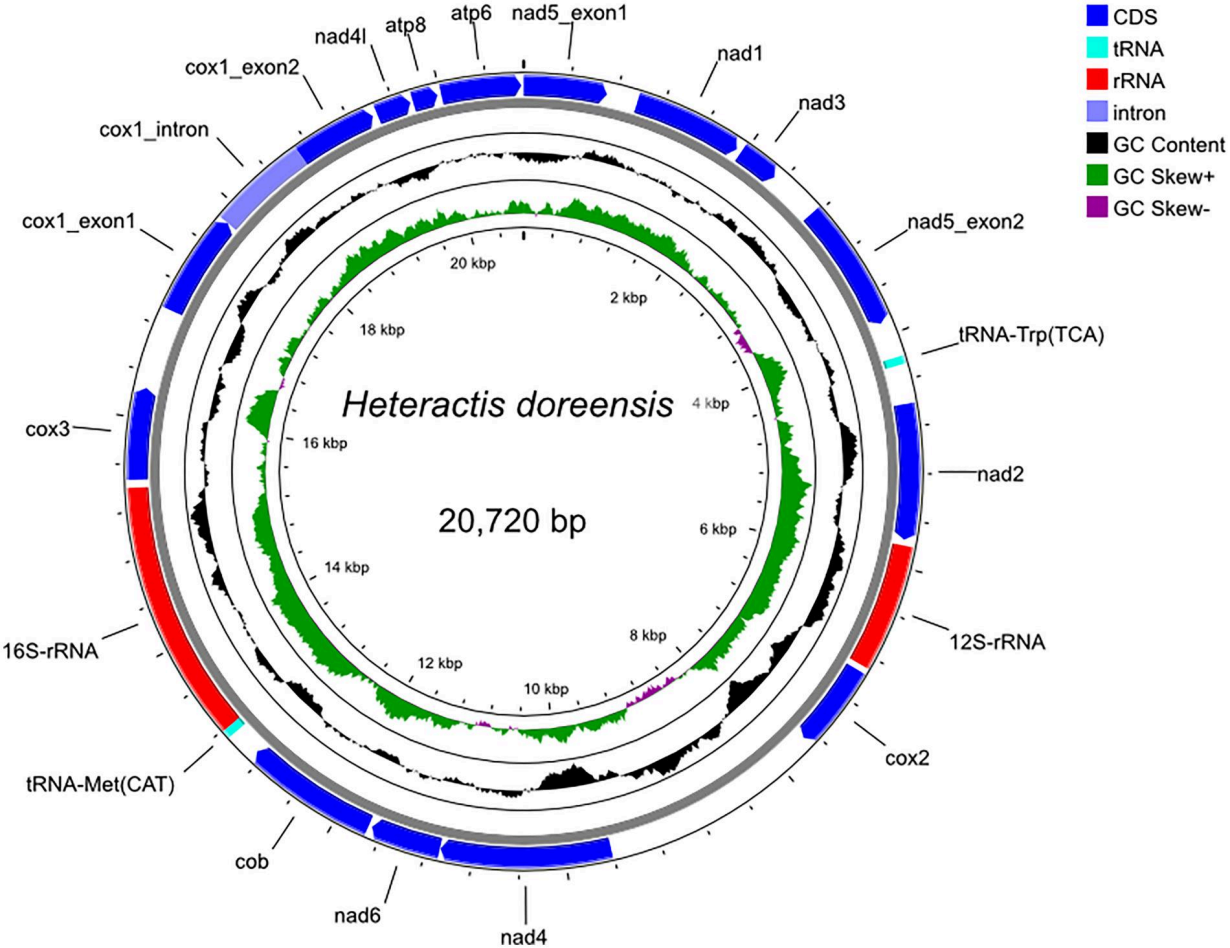
Mitochondrial genome map of *H. doreensis.* The complete mitochondrial genome of *H. doreensis* was 20,720 bp long and included 13 PCGs, two tRNA genes, and two rRNA genes. The overall base composition was as follows: 26.64% for A, 17.84% for C, 21.82% for G, and 33.71% for T. The outermost circle represents the gene name, direction of the encoding gene, and transcription; tRNA and rRNA have no direction. Inside the black circle is the GC proportion, and further inside is the GC offset. The innermost circle represents the length coordinate.

The mitochondrial genome of *H. doreensis* is a circular molecule of 20,720 bp in length (GenBank accession number: OP055896) (Supplementary Figure 2). Its overall nucleotide composition is 26.64% A, 17.84% C, 21.82% G, and 33.71% T, corresponding to GC and AT contents of 39.66% and 60.34%, respectively. The genome contains 13 protein-coding genes (PCGs), two tRNA genes (*tRNA^Trp^* and *tRNA^Met^*), two ribosomal RNA (rRNA) genes (*12S rRNA* and *16S rRNA*), and several non-coding regions (Supplementary Table 1). These genomic features collectively reflect the typical mitogenome architecture observed in Hexacorallia, with 13 PCGs spanning 11,847 bp, accounting for 57.18% of the total genome length. The *12S rRNA* gene (1081 bp) is located between nad1 and cox2, whereas the *16S rRNA* gene (1714 bp) is positioned between *tRNA^Met^* and cox3. All PCGs, except nad1, initiate with the ATG start codon. Termination codons vary, with nad2, nad5, and nad4l ending with TAG, whereas the remaining PCGs terminate with TAA (Figure 2). Furthermore, assembly metrics and inspection of terminal overlaps confirmed that the sequence was complete and circular, without any gaps or unresolved regions. The continuity between the terminal region (cox1-atp6) and the start region (nad5) further supports the circular organization of the mitogenome.

**Figure 3.**
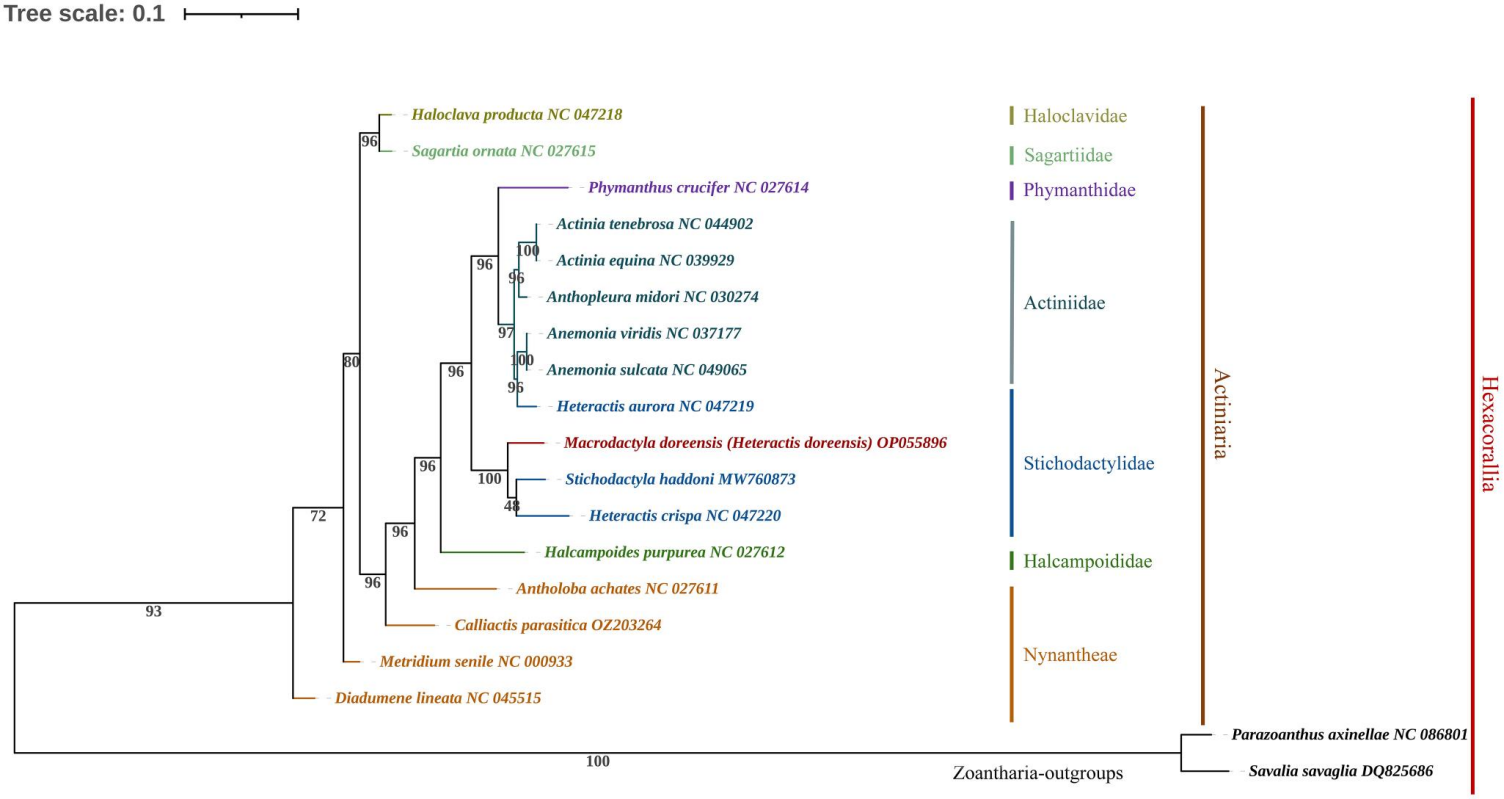
ML phylogenetic tree for *H. doreensis* and other Zoantharia species based on the complete mitochondrial genome. Bootstrap support values are indicated at each node. *Parazoanthus axinellae* and *Savalia savaglia*, both belonging to the order Zoantharia, were used as the outgroups. The dataset includes the following mitochondrial genomes: *Macrodactyla doreensis* (OP055896; this study name is *Heteractis doreensis*), *Actinia tenebrosa* (NC044902; Surm et al. 2019), *Actinia equina* (NC039929; Wilding and Weedall 2019), *Anthopleura midori* (NC030274; Zhang and Zhu 2017), *Anemonia sulcata* (NC049065; unpublished), *Anemonia viridis* (NC037177; Chi et al. 2018), *Halcampoides purpurea* (NC027612; Foox et al. 2016), *Haloclava producta* (NC047218; unpublished), *Metridium senile* (NC000933; Beagley et al. 1998), *Diadumene lineata* (NC045515; unpublished), *Calliactis parasitica* (OZ203264; unpublished), *Antholoba achates* (NC027611; Foox et al. 2016), *Phymanthus crucifer* (NC027614; Foox et al. 2016), *Sagartia ornata* (NC027615; Foox et al. 2016), *Stichodactyla haddoni* (MW760873; Johansen et al. 2021), *Heteractis crispa* (NC047220; unpublished), *Heteractis aurora* (NC047219; unpublished), *Savalia savaglia* (DQ825686; Sinniger et al. 2007), and *Parazoanthus axinellae* (NC086801; unpublished).

To investigate the evolutionary implications of the newly sequenced mitogenome, we performed a phylogenetic analysis. Phylogenetic reconstructions based on homologous mitochondrial markers indicate that the genus *Macrodactyla* comprises multiple divergent evolutionary lineages. Consequently, *Macrodactyla doreensis* was recently reclassified within the genus *Heteractis* based on strong nodal support (bootstrap > 90%) and shared synapomorphic characters (Hua et al. 2024). Accordingly, the specimen previously labeled as *M. doreensis* in Figure 3 is referred to as *H. doreensis*. We further analyzed the mitochondrial genome of *H. doreensis*, together with those of 18 additional Hexacorallia species, to infer relationships within the subclass. The resulting ML phylogeny revealed that *H. doreensis* clustered with other members of the genus *Heteractis*, which was supported by robust node support.

## 4. Discussion and conclusions

This study presents the first complete mitochondrial genome of *H. doreensis*, revealing the conservation of mitogenome structure and molecular characteristics. Previous research (Yap et al. 2023) demonstrated that the genus *Macrodactyla* is not monophyletic, as evidenced by mitochondrial (12S, 16S, cox3) and nuclear (28S) markers. Based on the complete mitochondrial genome of *H. doreensis*, our phylogenetic analysis aligns with these findings and further supports the reassignment of *M. doreensis* to the genus *Heteractis*. The ML tree reconstructed using MEGA11 places *H. doreensis* in a strongly supported clade (bootstrap = 96%) with *Stichodactyla haddoni* and *Heteractis crispa*, reinforcing its position within the family Stichodactylidae. This finding confirms the evolutionary distinction of *H. doreensis* and resolves long-standing taxonomic uncertainties surrounding this species (Figure 3). Currently, mitogenomic data for Actiniaria are limited, with substantial gaps in taxonomic representation across different families. This uneven sampling may challenge the accuracy of the phylogenetic framework by affecting branch support and topological stability at the deeper nodes of the tree. Therefore, expanding the mitogenomic dataset through further sequencing of underrepresented groups is essential for testing the robustness of the current phylogeny and uncovering broader evolutionary patterns in sea anemones. Our findings clarify the evolutionary position of *H. doreensis* within Stichodactylidae and contribute to the expansion of the mitochondrial genomic resources available for sea anemones. These resources not only enhance our understanding of the evolutionary history of this diverse group but also provide a more comprehensive foundation for future taxonomic and evolutionary studies within Actiniaria, facilitating comparative genomic research and advancing our understanding of the processes that shape their diversities.

## Supplementary Material

Supplementary Figure 1.jpg

Supplementary table 1.xls

## Data Availability

The genome sequence data that support the findings of this study are openly available in GenBank of NCBI at https://www.ncbi.nlm.nih.gov under the accession number OP055896. The associate BioProject, BioSample, and SRA numbers are PRJNA893408, SAMN31422303, and SRS15509380, respectively.
