# Supplementary figures and images for: Complete mitochondrial genome and phylogenetic analysis of the sea anemone Heteractis doreensis (Quoy & Gaimard, 1833)
Source: Mitochondrial DNA B Resour. 2026 Feb 9;11(3):388–92. doi: 10.1080/23802359.2026.2627019 (PMC12888344; doi:10.1080/23802359.2026.2627019)

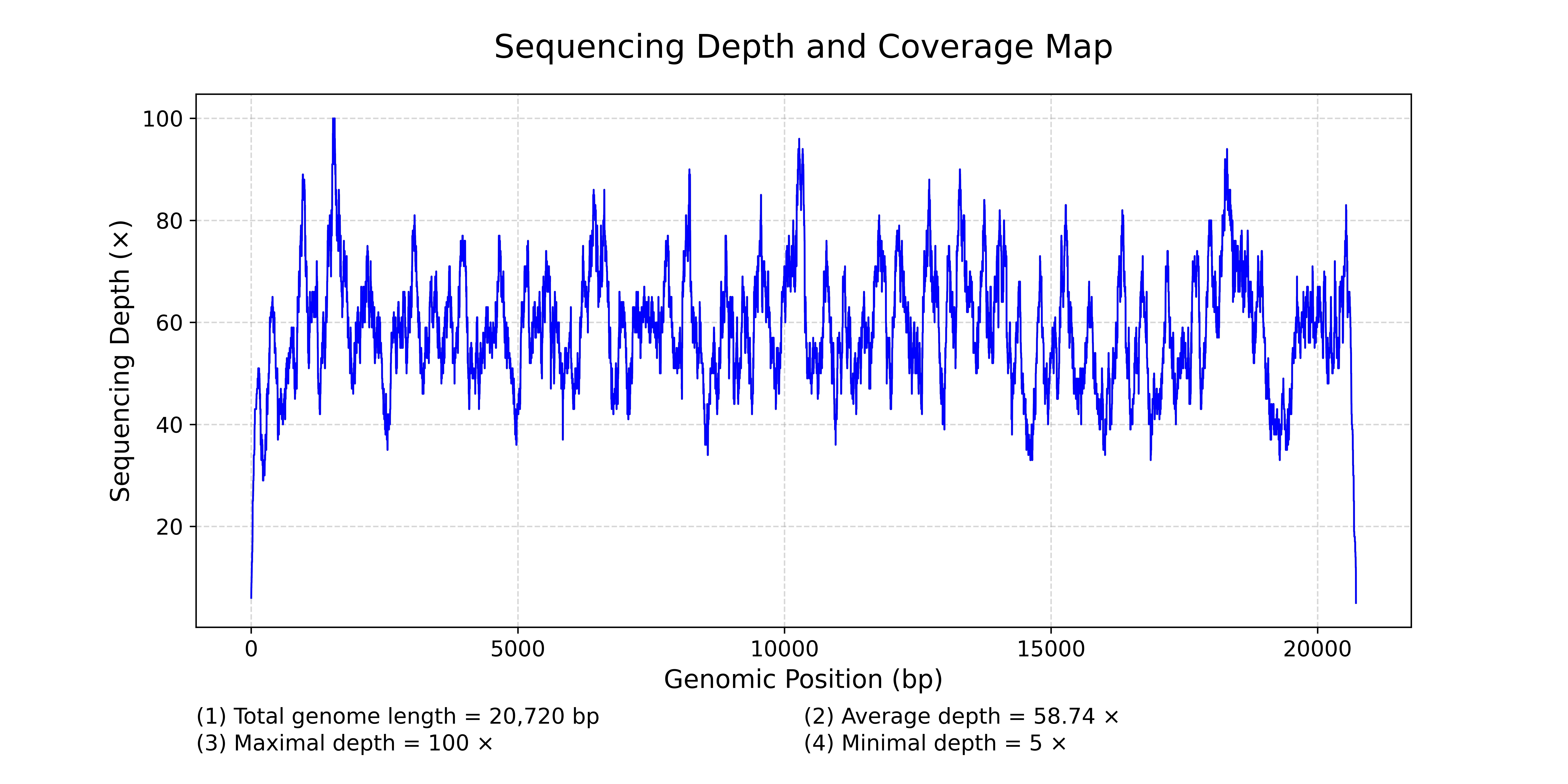

Supplement: Supplementary Figure 1.jpg [file TMDN_A_2627019_SM6328.jpg]
